# New Result on the Feedback Capacity of the Action-Dependent Dirty Paper Wiretap Channel

**DOI:** 10.3390/e23080929

**Published:** 2021-07-21

**Authors:** Guangfen Xie, Bin Dai

**Affiliations:** 1 School of Information Science and Technology, Southwest Jiaotong University, Chengdu 611756, China; gfxie@my.swjtu.edu.cn; 2 Peng Cheng Laboratory, Shenzhen 518055, China

**Keywords:** action encoder, channel feedback, dirty paper channel, intelligent reflecting surfaces, wiretap channel

## Abstract

The Gaussian wiretap channel with noncausal state interference available at the transmitter, which is also called the dirty paper wiretap channel (DP-WTC), has been extensively studied in the literature. Recently, it has been shown that taking actions on the corrupted state interference of the DP-WTC (also called the action-dependent DP-WTC) helps to increase the secrecy capacity of the DP-WTC. Subsequently, it has been shown that channel feedback further increases the secrecy capacity of the action-dependent DP-WTC (AD-DP-WTC), and a sub-optimal feedback scheme is proposed for this feedback model. In this paper, a two-step hybrid scheme and a corresponding new lower bound on the secrecy capacity of the AD-DP-WTC with noiseless feedback are proposed. The proposed new lower bound is shown to be optimal (achieving the secrecy capacity) and tighter than the existing one in the literature for some cases, and the results of this paper are further explained via numerical examples.

## 1. Introduction

Dirty paper coding is one of the most important pre-coding schemes in wireless communications and has a wide range of applications in information hiding [1]. Dirty paper coding was first investigated by Costa in his well-known paper on the dirty paper channel (DPC) [2], where a corrupted Gaussian state interference of a white Gaussian channel is noncausally known at the transmitter and not available at the receiver. Costa showed that the capacity of the DPC equals the capacity of the same model without state interference, which indicates that though the receiver does not know the state interference, it can still be perfectly removed by using dirty paper coding.

Note that in [2], the channel state is assumed to be generated by nature. However, in some practical scenarios, the state is affected or controlled by the communication systems, e.g., in intelligent reflecting surface-aided communication systems, the state is formed in part by the reflecting phase shift which is actually controlled by the transceiver [3]. Such a case was first investigated by Weissman in his paper on the action-dependent dirty paper channel (AD-DPC) [4], where the corrupted state interference of the DPC is affected and noncausally known by the transmitter. In [4], a lower bound on the capacity of AD-DPC was proposed, and the capacity was fully determined in [5].

In recent years, the study of the above dirty paper channels under additional secrecy constraints has received a lot attention. Specifically, [6] studied the discrete memoryless wiretap channel with state interference noncausally known by the transmitter, and obtained upper and lower bounds on the secrecy capacity. The authors of [7] extended the model studied in [6] to the broadcast situation, and also provided bounds on the secrecy capacity region of this extended model. The authors of [8,9] studied the Gaussian case of [6], namely, the dirty paper wiretap channel, and proposed bounds on the secrecy capacity. Furthermore, [8,9] pointed out that the proposed secret dirty paper coding increases the secrecy capacity of the Gaussian wiretap channel [10]. The above works mainly adopted the tools in [11,12] for establishing the secrecy rate/capacity. Very recently, the authors of [13] studied the AD-DPC with an additional eavesdropper, which is also called the action-dependent dirty paper wiretap channel (AD-DP-WTC), and proposed lower and upper bounds on the secrecy capacity. Note that the capacity results given in [9,13] indicate that:

- There is a penalty term between secrecy capacity and capacity of the same model without secrecy constraints.
- If the eavesdropper’s channel is less noisy than the legitimate receiver’s, the secrecy capacity may equal zero, i.e., no positive rate can be guaranteed for secure communications.

**Figure 1:**
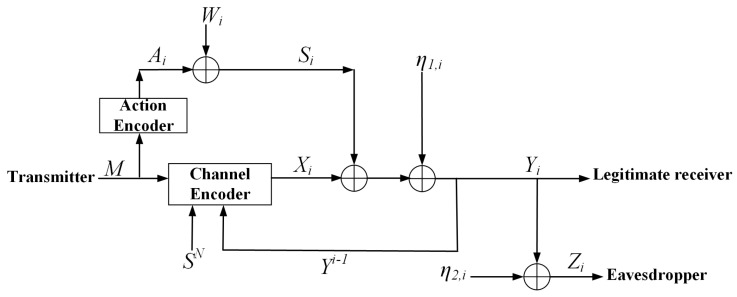
The AD-DP-WTC with noiseless feedback.

Very recently, it has been shown that channel feedback is an effective way to enhance the secrecy capacities of the DP-WTC [14,15] and the multi-input multi-output (MIMO) X-channels [16]. In [16], it has been shown that feedback increases the secure degrees of freedom (SDoF) region of the MIMO X-channel with secrecy constraints, which indicates that feedback also enlarges the secrecy capacity of the same model, even if the eavesdropper’s channel is less noisy than the legitimate receiver’s. The authors of [14,15,16] mainly adopted the idea of a generating secret key from the channel feedback and using this key to encrypt the transmitted message. Subsequently, [13] showed that a variation of the classical Schalkwijk–Kailath (SK) feedback scheme [17] for the point-to-point white Gaussian channel achieves the secrecy capacity of the DP-WTC with noiseless feedback, and the secrecy capacity equals the capacity of the same model without secrecy constraints, i.e., to achieve perfect secrecy, no rate needs to be sacrificed even if the eavesdropper gains advantage over the legitimate receiver. In addition, the authors of [13] also proposed a sub-optimal SK type feedback scheme for the AD-DP-WTC with noiseless feedback (see Figure 1), i.e., this scheme achieves a lower bound on the secrecy capacity of the AD-DP-WTC with noiseless feedback, and the secrecy capacity remains open.

In this paper, we derive a new lower bound on the secrecy capacity of the AD-DP-WTC with noiseless feedback, which is based on a hybrid two-step feedback scheme. The proposed new lower bound is shown to be optimal and tighter than the existing one in the literature for some cases, and the results of this paper are further explained via numerical examples. The remainder of this paper is organized as follows. A formal definition of the model studied in this paper and previous results are introduced in Section 2. The main result and the corresponding proof are given in Section 3 and Section 4, respectively. Section 5 includes the summary of all results in this paper and discusses future work.

## 2. Model Formulation and Previous Results

### 2.1. Model Formulation

For the AD-DP-WTC with noiseless feedback (see Figure 1), the *i*-th (i∈{1,2,…,N}) channel inputs and outputs are given by

(1) $$\begin{array}{c} S_{i}=A_{i}+W_{i},\;\;Y_{i}=X_{i}+S_{i}+\eta_{1,i},\;\;Z_{i}=Y_{i}+\eta_{2,i} \end{array}$$

where $X_{i}$ is the output of the channel encoder subject to an average power constraint *P* ($\frac{1}{N}\sum_{i=1}^{N}E\left[X_{i}^{2}\right]\leq P$), $A_{i}$ is the output of the action encoder subject to an average power constraint $P_{A}$ ($\frac{1}{N}\sum_{i=1}^{N}E\left[A_{i}^{2}\right]\leq P_{A}$), $Y_{i}$ and $Z_{i}$ are channel outputs, respectively, at the legitimate receiver and the eavesdropper, and $W_{i}$, $\eta_{1,i}$, $\eta_{2,i}$ are independent Gaussian noises and are independently identically distributed (i.i.d.) across the time index *i*. Note that $W_{i}∼N\left(0,\sigma_{w}^{2}\right)$, $\eta_{1,i}∼N\left(0,\sigma_{1}^{2}\right)$, and $\eta_{2,i}∼N\left(0,\sigma_{2}^{2}\right)$. The transmitted message *M* is uniformly drawn in its alphabet M={1,2,…,|M|}.

At time instant *i* (i∈{1,2,…,N}), a corrupted state interference $S_{i}$ is generated through a white Gaussian channel with i.i.d. noise $W_{i}∼N\left(0,\sigma_{w}^{2}\right)$ and channel input $A_{i}$, where $A_{i}$ is a (stochastic) function of the message *M*. Since the corrupted state interference $S^{N}=\left(S_{1},\ldots ,S_{N}\right)$ is noncausally known by the channel encoder, the *i*-th channel input $X_{i}$ is a (stochastic) function of the message *M*, $S^{N}$, and the feedback $Y^{i-1}=\left(Y_{1},Y_{2},\ldots ,Y_{i-1}\right)$.

The legitimate receiver generates an estimation $\hat{M}=\psi \left(Y^{N}\right)$, where ψ is the legitimate receiver’s decoding function, and the average decoding error probability equals

(2) $$P_{e}=\frac{1}{\left|M\right|}\sum_{m\in M_{1}}Pr\left\{\psi \left(Y^{N}\right)\neq m|m\;\;\mathrm{sent}\right\}.$$

The eavesdropper’s equivocation rate of the message *M* is denoted by

(3) $$\Delta =\frac{1}{N}H\left(M|Z^{N}\right).$$

A rate *R* is said to be achievable with perfect weak secrecy if for any ϵ and sufficiently large *N*, there exists a channel encoder–decoder such that

(4) $$\begin{array}{ccc} & & \frac{\log|M|}{N}=R,\;\;\Delta \geq R-\epsilon ,\;\;P_{e}\leq \epsilon . \end{array}$$

The *secrecy* capacity of the AD-DP-WTC with feedback, which is the maximum achievable secrecy rate defined in (4), is denoted by $C_{sag}^{f}$. A new lower bound, $R_{sag}^{f}$ on $C_{sag}^{f}$, will be given in the next section.

### 2.2. SK Feedback Scheme for the Point-to-Point White Gaussian Channel

For the point-to-point white Gaussian channel with feedback, at time *i* (i∈{1,2,…,N}), channel input and output are given by

(5) $$\begin{array}{ccc} & & Y_{i}=X_{i}+\eta_{i}, \end{array}$$

where $X_{i}$ is the channel input subject to an average power constraint *P* ($\frac{1}{N}\sum_{i=1}^{N}E\left[X_{i}^{2}\right]\leq P$), and $\eta_{i}∼N\left(0,\sigma^{2}\right)$ is the i.i.d. channel noise. The channel input $X_{i}$ is a function of the message *M* and the feedback $Y^{i-1}$. It is well known that the capacity $C_{g}^{f}$ of the white Gaussian channel with feedback equals the capacity $C_{g}$ of the same model without feedback, i.e.,

(6) $$\begin{array}{ccc} & & C_{g}^{f}=C_{g}=\frac{1}{2}\log\left(1+\frac{P}{\sigma^{2}}\right). \end{array}$$

The authors of [17] showed that the classical SK scheme achieves $C_{g}^{f}$, and this scheme is described below.

Since *M* takes the values in $M=\{1,2,\ldots ,2^{NR}\}$, we divide the interval [−0.5,0.5] into $2^{NR}$ equally spaced sub-intervals, and the center of each sub-interval is mapped to a message value in M. Let θ be the center of the sub-interval with respect to (w.r.t.) the message *M*.

At time 1, the transmitter transmits

(7) $$X_{1}=\sqrt{12P}\theta .$$

The receiver receives $Y_{1}=X_{1}+\eta_{1}$, and obtains an estimation of θ by computing

(8) $${\hat{\theta }}_{1}=\frac{Y_{1}}{\sqrt{12P}}=\theta +\frac{\eta_{1}}{\sqrt{12P}}=\theta +\epsilon_{1},$$

where $\epsilon_{1}={\hat{\theta }}_{1}-\theta =\frac{\eta_{1}}{\sqrt{12P}}$, and $\alpha_{1}≜\frac{\sigma^{2}}{12P}$.

At time *k* (2≤k≤N), the receiver obtains $Y_{k}=X_{k}+\eta_{k}$, and obtains an estimation of $\theta_{k}$ by computing

(9) $${\hat{\theta }}_{k}={\hat{\theta }}_{k-1}-\frac{E[Y_{k}\epsilon_{k-1}]}{E[Y_{k}^{2}]}Y_{k},$$

where

(10) $$\epsilon_{k}={\hat{\theta }}_{k}-\theta ,$$

(10) yields that

(11) $$\epsilon_{k}=\epsilon_{k-1}-\frac{E[Y_{k}\epsilon_{k-1}]}{E[Y_{k}^{2}]}Y_{k}.$$

At time *k* (k∈{2,3,…,N}), the transmitter sends

(12) $$X_{k}=\sqrt{\frac{P}{\alpha_{k-1}}}\epsilon_{k-1},$$

where $\alpha_{k-1}≜Var\left(\epsilon_{k-1}\right)$.

In [17], it has been shown that the decoding error $P_{e}$ of the above coding scheme is upper bounded by

(13) $$P_{e}\leq Pr\{|\epsilon_{N}\left|>\frac{1}{2|M|-1}\right\}\leq 2Q\left(\frac{1}{2\cdot 2^{NR}}\frac{1}{\sqrt{\alpha_{N}}}\right),$$

where Q(x) is the tail of the unit Gaussian distribution evaluated at *x*, and

(14) $$\alpha_{N}=\frac{\sigma^{2}}{12P}{\left(\frac{\sigma^{2}}{P+\sigma^{2}}\right)}^{N-1}.$$

From (13) and (14), we conclude that if

(15) $$R<\frac{1}{2}\log\left(1+\frac{P}{\sigma^{2}}\right)=C_{g}^{f},$$

$P_{e}\to 0$ as N→∞. Recently, [18] showed that the above classical SK scheme, which is not designed with the consideration of secrecy, already achieves the secrecy capacity of the Gaussian wiretap channel with noiseless feedback, i.e., the corresponding secrecy capacity equals the capacity $C_{g}^{f}$ of the same model without secrecy constraints.

### 2.3. Previous Results on the AD-DP-WTC with Feedback

For the capacity $C_{sag}^{f}$ of the AD-DP-WTC with feedback, [13] showed that it is bounded by

(16) $$\begin{array}{ccc} & & L\leq C_{sag}^{f}\leq C_{ag}, \end{array}$$

where

(17) $$\begin{array}{ccc} & & C_{ag}=\max_{\left(\rho_{1},\rho_{2}\right):\rho_{1}^{2}+\rho_{2}^{2}\leq 1}\frac{1}{2}\log\left(1+\frac{P(1-\rho_{1}^{2}-\rho_{2}^{2})}{\sigma_{1}^{2}}\right)\\ & & +\frac{1}{2}\log\left(1+\frac{{\left(\sqrt{P_{A}}+\rho_{2}\sqrt{P}\right)}^{2}}{P\left(1-\rho_{1}^{2}-\rho_{2}^{2}\right)+{\left(\sigma_{w}+\rho_{1}\sqrt{P}\right)}^{2}+\sigma_{1}^{2}}\right), \end{array}$$

(18) $$\begin{array}{ccc} & & L=\max_{\left(\rho_{1},\rho_{2}\right):\rho_{1}^{2}+\rho_{2}^{2}\leq 1}\frac{1}{2}\log\left(1+\frac{{\left(\sqrt{P_{A}}+\rho_{2}\sqrt{P}\right)}^{2}}{P\left(1-\rho_{1}^{2}-\rho_{2}^{2}\right)+{\left(\sigma_{w}+\rho_{1}\sqrt{P}\right)}^{2}+\sigma_{1}^{2}}\right). \end{array}$$

Note that $C_{ag}$ is the capacity of the AD-DPC without feedback, and is given in [5].

**Proof Sketch of** **(16).** Since the capacity $C_{sag}^{f}$ of the AD-DP-WTC with feedback is no larger than that of the same model without secrecy constraints, and feedback does not increase the capacity of the AD-DPC [4], and $C_{ag}$ serves as a trivial upper bound on $C_{sag}^{f}$. The lower bound $C_{sag}^{f}\geq L$ is derived by constructing

(19) $$\begin{array}{ccc} & & X=\frac{\rho_{2}^{*}\sqrt{P}}{\sqrt{P_{A}}}A+\frac{\rho_{1}^{*}\sqrt{P}}{\sigma_{w}}W, \end{array}$$

for $\rho_{1}^{*2}+\rho_{2}^{*2}=1$, and

(20) $$\begin{array}{ccc} & & X=\frac{\rho_{2}^{*}\sqrt{P}}{\sqrt{P_{A}}}A+\frac{\rho_{1}^{*}\sqrt{P}}{\sigma_{w}}W+G, \end{array}$$

for $\rho_{1}^{*2}+\rho_{2}^{*2}<1$, where *G* is randomly generated according to $G∼N(0,P\left(1-\rho_{1}^{*2}-\rho_{2}^{*2}\right))$ and it is independent of *A* and *W*. Substituting (19) and (20) into (1), the AD-DP-WTC with feedback is equivalent to the Gaussian wiretap channel with feedback with input *A*, channel noise $W+G+\eta_{1}+\eta_{2}$, channel output *Y* of the legitimate receiver, and the channel output *Z* of the eavesdropper. Directly applying the SK scheme introduced in the preceding subsection, we conclude that *L* is achievable. Moreover, from [18], we know that the SK scheme also achieves the secrecy capacity of the Gaussian wiretap channel with noiseless feedback, which indicates that *L* is achievable with perfect weak secrecy, and the proof is completed. □

Note that the above lower and upper bounds on $C_{sag}^{f}$ do not meet in general, and exploring a tighter lower bound on $C_{sag}^{f}$ is the motivation of this paper.

## 3. Main Results of this Paper

### 3.1. A New Lower Bound on the Secrecy Capacity of the AD-DP-WTC with Feedback

**Theorem** **1.** *A new lower bound on the secrecy capacity $C_{sag}^{f}$ of the AD-DP-WTC with feedback is given by*

(21) $$\begin{array}{ccc} & & R_{sag}^{f}=\max_{\left(\rho_{1},\rho_{2}\right):\rho_{1}^{2}+\rho_{2}^{2}\leq 1}\left\{\frac{1}{2}\log\left(1+\frac{(1-\rho_{1}^{2}-\rho_{2}^{2})P}{\sigma_{1}^{2}}\right)\right.\\ & & +\frac{1}{2}\log\left(1+\frac{{\left(\sqrt{P_{A}}+\rho_{2}\sqrt{P}\right)}^{2}}{P\left(1-\rho_{1}^{2}-\rho_{2}^{2}\right)+{\left(\sigma_{w}+\rho_{1}\sqrt{P}\right)}^{2}+\sigma_{1}^{2}}\right)\\ & & \left.-\frac{1}{2}\log\left(1+\frac{{\left(\sqrt{P_{A}}+\rho_{2}\sqrt{P}\right)}^{2}}{P\left(1-\rho_{1}^{2}-\rho_{2}^{2}\right)+{\left(\sigma_{w}+\rho_{1}\sqrt{P}\right)}^{2}+\sigma_{1}^{2}+\sigma_{2}^{2}}\right)\right\}. \end{array}$$

**Remark** **1.** *Comparing the new lower bound in (21) with the upper bound $C_{ag}$ in (16), it is easy to see that there still exists a gap between the two bounds due to the penalty term $\frac{1}{2}\log\left(1+\frac{{\left(\sqrt{P_{A}}+\rho_{2}\sqrt{P}\right)}^{2}}{P\left(1-\rho_{1}^{2}-\rho_{2}^{2}\right)+{\left(\sigma_{w}+\rho_{1}\sqrt{P}\right)}^{2}+\sigma_{1}^{2}+\sigma_{2}^{2}}\right)$.*

The following *Corollary 1* shows that the proposed new lower bound in (21) is optimal for a special case. Moreover, the following *Corollary 2* shows that the new lower bound in (21) is tighter than the existing lower bound in (16) when $\sigma_{w}^{2}$ tends to infinity.

**Corollary** **1.** *For $\sigma_{2}^{2}\to \infty$,*

(22) $$\begin{array}{ccc} & & \lim_{\sigma_{2}^{2}\to \infty }R_{sag}^{f}=C_{ag}, \end{array}$$

*which indicates that the secrecy capacity is determined for this case.*

**Proof.**  Formula (22) is directly obtained by using (21) and letting $\sigma_{2}^{2}\to \infty$. Hence the proof is completed. □

**Corollary** **2.** (23) $$\begin{array}{ccc} & & \lim_{\sigma_{w}^{2}\to \infty }R_{sag}^{f}\geq \lim_{\sigma_{w}^{2}\to \infty }L, \end{array}$$

*where L is the existing lower bound defined in (18).*

**Proof.**  Define the gap $R_{G}$ between the new lower bound in (21) and the existing lower bound in (18) by

(24) $$\begin{array}{ccc} & & R_{G}=R_{sag}^{f}-L=\max_{\left(\rho_{1},\rho_{2}\right):\rho_{1}^{2}+\rho_{2}^{2}\leq 1}\left\{\frac{1}{2}\log\left(1+\frac{(1-\rho_{1}^{2}-\rho_{2}^{2})P}{\sigma_{1}^{2}}\right)\right.\\ & & \left.-\frac{1}{2}\log\left(1+\frac{{\left(\sqrt{P_{A}}+\rho_{2}\sqrt{P}\right)}^{2}}{P\left(1-\rho_{1}^{2}-\rho_{2}^{2}\right)+{\left(\sigma_{w}+\rho_{1}\sqrt{P}\right)}^{2}+\sigma_{1}^{2}+\sigma_{2}^{2}}\right)\right\}. \end{array}$$

- For the case that the maximum is achieved when $\rho_{1}^{2}+\rho_{2}^{2}=1$,
  (25) $$\begin{array}{ccc} & & \lim_{\sigma_{w}^{2}\to \infty }R_{G}=0. \end{array}$$
- For the case that the maximum is achieved when $\rho_{1}^{2}+\rho_{2}^{2}<1$,
  (26) $$\begin{array}{ccc} & & \lim_{\sigma_{w}^{2}\to \infty }R_{G}>0. \end{array}$$

Combining (25) and (26), we conclude that $\lim_{\sigma_{w}^{2}\to \infty }R_{G}\geq 0$, which indicates that $\lim_{\sigma_{w}^{2}\to \infty }$ $R_{sag}^{f}\geq \lim_{\sigma_{w}^{2}\to \infty }L$. The proof of *Corollary 2* is completed. □

**Figure 2:**
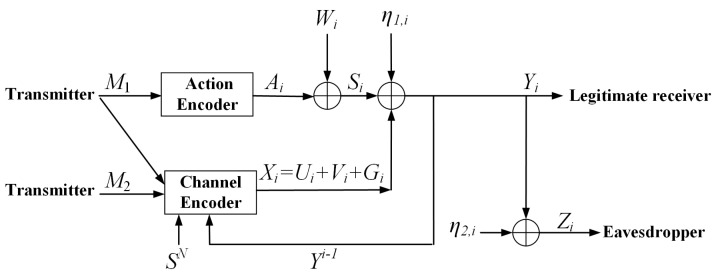
The new coding scheme of AD-DP-WTC with noiseless feedback.

**Figure 3:**
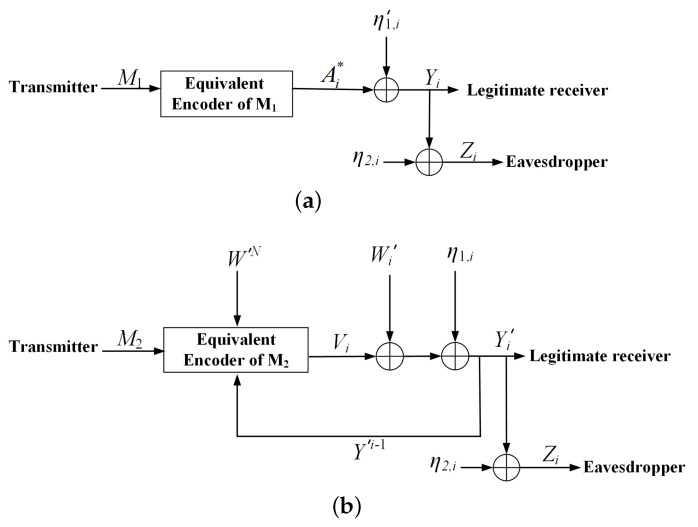
Equivalentcoding scheme. (**a**) The equivalent channel model of the sub-message $M_{1}$. (**b**) The equivalent channel model of the sub-message $M_{2}$.

**Proof Sketch of Theorem 1.**  The main idea of the achievable scheme is briefly illustrated by Figure 2 and Figure 3. In Figure 2, we split the message *M* into two parts $M=(M_{1},M_{2})$, where the sub-message $M_{j}$(j=1,2) is uniformly distributed in $M_{j}=\left\{1,\ldots ,2^{NR_{j}}\right\}$. The sub-message $M_{1}$ is available at both the action encoder and channel encoder, and the sub-message $M_{2}$ is only available at the channel encoder. Let $X_{i}=U_{i}+V_{i}+G_{i}$, where $M_{1}$ is encoded as $U_{i}$ with power $\rho_{2}^{2}P$, $M_{2}$ is encoded as $V_{i}$ with power $P^{\prime }=\left(1-\rho_{1}^{2}-\rho_{2}^{2}\right)P$, $G_{i}=\sqrt{\frac{\rho_{1}^{2}P}{\sigma_{w}^{2}}}W_{i}$, and $-1\leq \rho_{j}\leq 1$ for j=1,2. Moreover, $M_{1}$ is also encoded as $A_{i}$ with power $P_{A}$, and

(27) $$\begin{array}{ccc} & & U_{i}=\sqrt{\frac{\rho_{2}^{2}P}{P_{A}}}A_{i}, \end{array}$$

which indicates that $U_{i}$ is a deterministic function of $A_{i}$. □

The *i*-th (i∈{1,2,…,N}) channel inputs and outputs are rewritten as

(28) $$\begin{array}{ccc} & & Y_{i}=X_{i}+S_{i}+\eta_{1,i}=U_{i}+V_{i}+G_{i}+A_{i}+W_{i}+\eta_{1,i}=A_{i}^{*}+V_{i}+W_{i}^{\prime }+\eta_{1,i}\\ & & Z_{i}=Y_{i}+\eta_{2,i}, \end{array}$$

where $W_{i}^{\prime }=G_{i}+W_{i}$ is an i.i.d Gaussian noise process with zero mean and variance ${\sigma_{w}^{\prime }}^{2}={\left(\sigma_{w}+\sqrt{\rho_{1}^{2}P}\right)}^{2}$, and $A_{i}^{*}=A_{i}+U_{i}$ is subject to an average power constraint $P^{*}=P_{A}+\rho_{2}^{2}P+2\sqrt{\rho_{2}^{2}PP_{A}}$.

In Figure 3, since $M_{1}$ is known by the channel encoder, the codeword $A_{i}^{*}=A_{i}+U_{i}$ can be subtracted when applying an SK type feedback scheme to $M_{2}$, i.e., the transmission of $M_{2}$ is through an equivalent channel with input $V_{i}$, output

(29) $$\begin{array}{c} Y_{i}^{\prime }=Y_{i}-A_{i}^{*}=V_{i}+W_{i}^{\prime }+\eta_{1,i}. \end{array}$$

Moreover, since $M_{1}$ and $S^{N}$ are known by the channel encoder, $W^{N}=S^{N}-A^{N}$ and $G^{N}=\sqrt{\frac{\rho_{1}^{2}P}{\sigma_{w}^{2}}}W^{N}$, $W^{{}^{\prime }N}=W^{N}+G^{N}$ can be viewed as state interference which is noncausally known at the encoder of the equivalent channel of $M_{2}$ (namely, the equivalent encoder of $M_{2}$).

In addition, the transmission of $M_{1}$ is through an equivalent channel with inputs $A_{i}^{*}=A_{i}+U_{i}$, output $Y_{i}=A_{i}^{*}+V_{i}+W_{i}^{\prime }+\eta_{1,i}$, and channel noise $\eta_{1,i}^{\prime }=V_{i}+W_{i}^{\prime }+\eta_{1,i}$, which is nonwhite Gaussian noise since $V_{i}$ is not i.i.d generated.

Then, applying an SK type scheme to $M_{2}$, and Wyner’s random binning scheme [11] together with Feinstein’s greedy coding scheme [19] to $M_{1}$, the lower bound in *Theorem 1* is obtained. The detail of the proof is given in the next section.

### 3.2. Numerical Results

**Figure 4:**
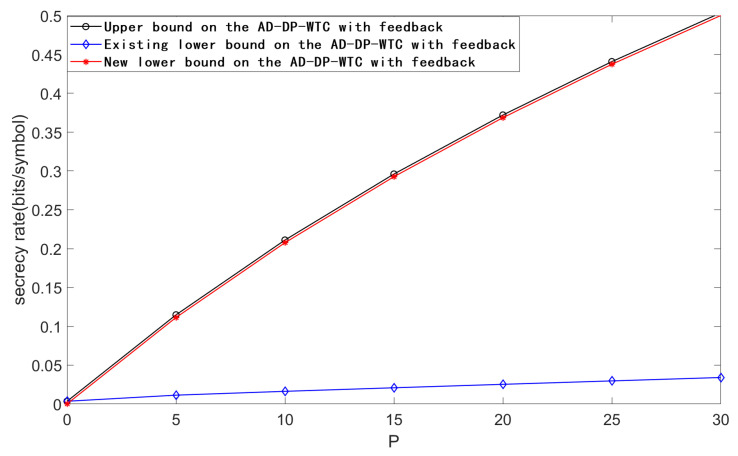
Comparison of the bounds on the secrecy capacity of AD-DP-WTC with feedback for $P_{A}=10$, $\sigma_{w}^{2}=2000$, $\sigma_{1}^{2}=30$, $\sigma_{2}^{2}=100$, and *P* taking values in [0,30].

Figure 4 plots the upper bound, the existing lower bound and the new lower bound on the secrecy capacity of the AD-DP-WTC with feedback for $P_{A}=10$, $\sigma_{w}^{2}=2000$, $\sigma_{1}^{2}=30$, $\sigma_{2}^{2}=100$, and *P* taking values in [0,30]. From Figure 4, we see that our new scheme performs better than the existing one when the noise variance $\sigma_{w}^{2}$ is large enough.

**Figure 5:**
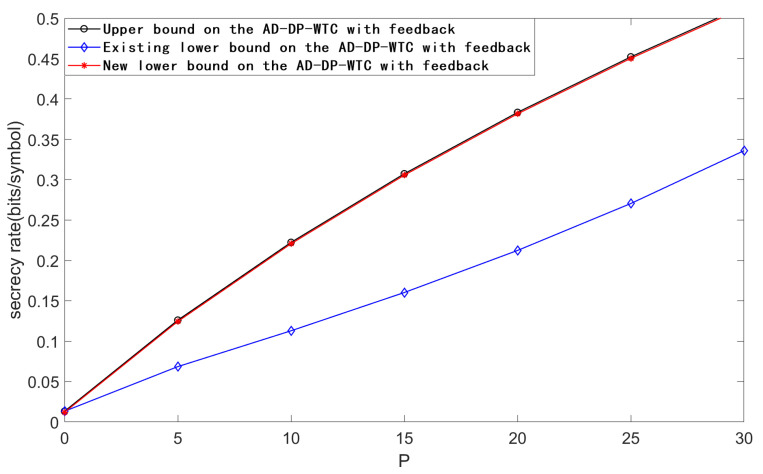
Comparison of the bounds on the secrecy capacity of AD-DP-WTC with feedback for $P_{A}=5$, $\sigma_{w}^{2}=240$, $\sigma_{1}^{2}=30$, $\sigma_{2}^{2}=2500$, and *P* taking values in [0,30].

Figure 5 plots the bounds for $P_{A}=5$, $\sigma_{w}^{2}=240$, $\sigma_{1}^{2}=30$, $\sigma_{2}^{2}=2500$, and *P* taking values in [0,30]. From Figure 5, we see that our new lower bound almost meets the upper bound when the eavesdropper’s channel noise variance $\sigma_{2}^{2}$ is large enough.

## 4. Proof of Theorem 1

The encoding and decoding procedure of Figure 3 is described below. Since $M_{2}$ takes values in $M_{2}\in \left\{1,2,\ldots ,2^{NR_{2}}\right\}$, the interval [−0.5,0.5] is divided into $2^{NR_{2}}$ equally spaced sub-intervals, and the center of each sub-interval is mapped to a message value. Let θ be the center of the sub-interval w.r.t. the message $M_{2}$ (the variance of θ approximately equals $\frac{1}{12}$ ). Let $A^{*N}=\left(A_{1}^{*}=0,A_{2}^{*},\ldots ,A_{N}^{*}\right)=\left(0,A_{2}^{*N}\right)$, where $A_{2}^{*N}=\left(A_{2}^{*},\ldots ,A_{N}^{*}\right)$. Here, $A_{2}^{*N}$ is the codeword of the sub-message $M_{1}$ and a dummy message $M_{1}^{*}$, and it is generated by Feinstein’s greedy construction for the non-i.i.d. Gaussian channel [19]. Moreover, $M_{1}$ and $M_{1}^{*}$ are uniformly distributed in $\left\{1,\ldots ,2^{\left(N-1\right)R_{1}}\right\}$ and $\left\{1,\ldots ,2^{\left(N-1\right)R^{*}}\right\}$, respectively.

*Encoding procedure*: Before the transmission, for a given sub-message $M_{1}$, first, a dummy message $M_{1}^{*}$ is randomly chosen from its alphabet $\left\{1,\ldots ,2^{\left(N-1\right)R^{*}}\right\}$. Then, Feinstein’s greedy construction [19] is applied to encode the message pair $\left(M_{1},M_{1}^{*}\right)$ as the codeword $A_{2}^{*N}=\left(A_{2}^{*},\ldots ,A_{N}^{*}\right)$.

At time 1, the equivalent encoders of $M_{1}$ and $M_{2}$, respectively, send

(30) $$\begin{array}{c} A_{1}^{*}=0,\;\;V_{1}=\sqrt{12P^{{}^{\prime }}}\left(\theta -\frac{W_{1}^{{}^{\prime }}}{\sqrt{12P^{{}^{\prime }}}}+B\right), \end{array}$$

where

(31) $$\begin{array}{c} B=\sum_{i=2}^{N}\frac{E(Y_{i}^{\prime \prime }\varepsilon_{i-1})}{E{\left(Y_{i}^{\prime \prime }\right)}^{2}}W_{i}^{{}^{\prime }},\;\;\;P^{\prime }=\left(1-\rho_{1}^{2}-\rho_{2}^{2}\right)P,\;\;\;Y_{i}^{\prime \prime }=V_{i}+\eta_{1,i} \end{array}$$

and $\varepsilon_{i-1}$ will be defined later.

At time 2, once receiving the feedback $Y_{1}=V_{1}+W_{1}^{{}^{\prime }}+\eta_{1,1}$, the equivalent encoder of $M_{2}$ computes

(32) $$\begin{array}{ccc} & & \frac{Y_{1}}{\sqrt{12P^{\prime }}}=\theta +B+\frac{\eta_{1,1}}{\sqrt{12P^{\prime }}}=\theta +B+\varepsilon_{1}, \end{array}$$

where $\varepsilon_{1}=\frac{\eta_{1,1}}{\sqrt{12P^{\prime }}}$ and $\alpha_{1}=Var\left(\varepsilon_{1}\right)$. The equivalent encoders of $M_{1}$ and $M_{2}$, respectively, send $A_{2}^{*}$ (the first component of $A_{2}^{*N}$) and

(33) $$\begin{array}{c} V_{2}=\sqrt{\frac{P^{\prime }}{\alpha_{1}}}\varepsilon_{1}. \end{array}$$

At time *k* (3≤k≤N), once receiving the feedback $Y_{k-1}=A_{k-1}^{*}+V_{k-1}+\eta_{1,k-1}+W_{k-1}^{{}^{\prime }}$, the equivalent encoder of $M_{2}$ computes

(34) $$\begin{array}{ccc} & & \varepsilon_{k-1}=\varepsilon_{k-2}-\frac{E(Y_{k-1}^{{}^{\prime \prime }}\varepsilon_{k-2})}{E{\left(Y_{k-1}^{{}^{\prime \prime }}\right)}^{2}}Y_{k-1}^{{}^{\prime \prime }}, \end{array}$$

where $Y_{k-1}^{\prime \prime }=Y_{k-1}-A_{k-1}^{*}-W_{k-1}^{{}^{\prime }}=V_{k-1}+\eta_{1,k-1}$ and $\alpha_{k-1}=Var\left(\varepsilon_{k-1}\right)$. The equivalent encoders of $M_{1}$ and $M_{2}$, respectively, send $A_{k}^{*}$ (the k−1-th component of $A_{2}^{*N}$) and

(35) $$\begin{array}{c} V_{k}=\sqrt{\frac{P^{\prime }}{\alpha_{k-1}}}\varepsilon_{k-1}. \end{array}$$

**Lemma** **1.** *For 3≤k≤N, the general terms of $\alpha_{k-1}$ and $V_{k}$ can be expressed as*

(36) $$\begin{array}{ccc} & & \alpha_{k-1}=Var\left(\varepsilon_{k-1}\right)=\alpha_{1}{\left(\frac{\sigma_{1}^{2}}{P^{\prime }+\sigma_{1}^{2}}\right)}^{k-2}, \end{array}$$

(37) $$\begin{array}{ccc} & & V_{k}=\frac{1}{\sqrt{12\alpha_{k-1}}}{\left(\frac{\sigma_{1}^{2}}{P^{\prime }+\sigma_{1}^{2}}\right)}^{k-2}\eta_{1,1}-\sum_{i=1}^{k-2}\sqrt{\frac{\alpha_{i}}{\alpha_{k-1}}}\frac{P^{\prime }{\left(\sigma_{1}^{2}\right)}^{k-2-i}}{{\left(P^{\prime }+\sigma_{1}^{2}\right)}^{k-(i+1)}}\eta_{1,i+1}. \end{array}$$

**Proof.**  The proof of *Lemma 1* is in Appendix A. □

*Decoding procedure*: The legitimate receiver does a two-step decoding scheme. First, by applying Feinstein’s decoding rule [19] to the decoding of $A_{2}^{*N}$. According to Feinstein’s lemma [19], the decoding error probability of $M_{1}$ and $M_{1}^{*}$, denoted by $P_{e1}$, can be arbitrarily small if

(38) $$\begin{array}{c} R_{1}+R^{*}\leq \frac{I(A_{2}^{*N};Y_{2}^{N})}{N-1}, \end{array}$$

where $Y_{2}^{N}=\left(Y_{2},Y_{3},\ldots ,Y_{N}\right)$.

Second, after decoding $M_{1}$, the legitimate receiver obtains $A_{k}^{*}$ for all 2≤k≤N, and subtracts $A_{k}^{*}$ from $Y_{k}$, then the legitimate receiver obtains $Y_{k}^{{}^{\prime }}=Y_{k}-A_{k}^{*}=V_{k}+\eta_{1,k}+W_{k}^{{}^{\prime }}$.

At time 1, the legitimate receiver obtains $Y_{1}=V_{1}+W_{1}^{{}^{\prime }}+\eta_{1,1}$ and computes

(39) $$\begin{array}{c} {\hat{\theta }}_{1}=\frac{Y_{1}}{\sqrt{12P^{{}^{\prime }}}}=\theta +B+\varepsilon_{1}. \end{array}$$

At time *k* (2≤k≤N), the legitimate receiver’s estimation ${\hat{\theta }}_{k}$ of θ is given by

(40) $$\begin{array}{ccc} & & {\hat{\theta }}_{k}={\hat{\theta }}_{k-1}-\frac{E(Y_{k}^{{}^{\prime \prime }}\varepsilon_{k-1})}{E{\left(Y_{k}^{{}^{\prime \prime }}\right)}^{2}}Y_{k}^{{}^{\prime }}\\ & & \overset{\left(a\right)}{=}{\hat{\theta }}_{k-1}+\varepsilon_{k}-\varepsilon_{k-1}-\frac{E(Y_{k}^{{}^{\prime \prime }}\varepsilon_{k-1})}{E{\left(Y_{k}^{{}^{\prime \prime }}\right)}^{2}}W_{k}^{{}^{\prime }}\\ & & ={\hat{\theta }}_{1}+\varepsilon_{k}-\varepsilon_{1}-\sum_{j=2}^{k}\frac{E(Y_{j}^{{}^{\prime \prime }}\varepsilon_{j-1})}{E{\left(Y_{j}^{{}^{\prime \prime }}\right)}^{2}}W_{j}^{{}^{\prime }}\\ & & \overset{\left(b\right)}{=}\theta +\varepsilon_{k}+B-\sum_{j=2}^{k}\frac{E(Y_{j}^{{}^{\prime \prime }}\varepsilon_{j-1})}{E{\left(Y_{j}^{{}^{\prime \prime }}\right)}^{2}}W_{j}^{{}^{\prime }}, \end{array}$$

where $Y_{k}^{{}^{\prime }}=V_{k}+\eta_{1,k}+W_{k}^{{}^{\prime }},\;\;\;Y_{k}^{{}^{\prime \prime }}=V_{k}+\eta_{1,k}$, (*a*) follows from (34), and (*b*) follows from (39). From (40), we can conclude that for k=N,

(41) $$\begin{array}{ccc} & & {\hat{\theta }}_{N}=\theta +\varepsilon_{N}+B-\sum_{j=2}^{N}\frac{E(Y_{j}^{{}^{\prime \prime }}\varepsilon_{j-1})}{E{\left(Y_{j}^{{}^{\prime \prime }}\right)}^{2}}W_{j}^{{}^{\prime }}\\ & & \overset{\left(c\right)}{=}\theta +\varepsilon_{N}, \end{array}$$

where (*c*) follows from (31).

Now we bound the decoding error probability $P_{e2}$ of $M_{2}$ as follows. From ${\hat{\theta }}_{N}=\theta +\varepsilon_{N}$ and the definition of θ, we have

(42) $$\begin{array}{ccc} & & P_{e2}\leq Pr[|\varepsilon_{N}\left|\geq \frac{1}{2(|M_{2}|-1)}\right]\\ & & \overset{\left(d\right)}{\leq }2Q\left(\frac{1}{2\times 2^{NR_{2}}\sqrt{\alpha_{N}}}\right)\\ & & \overset{\left(e\right)}{=}2Q\left(\frac{1}{2\sqrt{\frac{\sigma_{1}^{2}+P^{\prime }}{12P^{\prime }}}}2^{-NR_{2}}2^{\frac{N}{2}\log\left(1+\frac{P^{\prime }}{\sigma_{1}^{2}}\right)}\right)\\ & & =2Q\left(\frac{1}{2\sqrt{\frac{\sigma_{1}^{2}+P^{\prime }}{12P^{\prime }}}}2^{N(\frac{1}{2}\log\left(1+\frac{P^{\prime }}{\sigma_{1}^{2}}\right)-R_{2})}\right), \end{array}$$

where (d) following from Q(x) is the tail of the unit Gaussian distribution evaluated at *x*, and (e) is from Lemma 1. Since Q(x) is decreasing while *x* is increasing, from (42), we can conclude that if

(43) $$\begin{array}{c} R_{2}\leq \frac{1}{2}\log\left(1+\frac{P^{\prime }}{\sigma_{1}^{2}}\right)=\frac{1}{2}\log\left(1+\frac{(1-\rho_{1}^{2}-\rho_{2}^{2})P}{\sigma_{1}^{2}}\right), \end{array}$$

$P_{e2}\to 0$ as *N* is large enough.

Note that the total decoding error probability $P_{e}$ of $M=(M_{1},M_{2})$ is upper bounded by $P_{e}\leq P_{e1}+P_{e2}$. From the above analysis, we conclude that $P_{e}\to 0$ as *N* tends to infinity if (38) and (43) are guaranteed.

*Equivocation analysis*: We bound the equivocation rate $\frac{H(M|Z^{N})}{N}$ as follows:

(44) $$\begin{array}{c} \frac{H(M|Z^{N})}{N}=\frac{H(M_{1},M_{2}|Z^{N})}{N}=\frac{H\left(M_{1}|Z^{N}\right)+H\left(M_{2}|Z^{N},M_{1}\right)}{N}. \end{array}$$

The first part $\frac{H(M_{1}|Z^{N})}{N}$ of (44) is bounded by

(45) $$\begin{array}{ccc} & & \frac{H(M_{1}|Z^{N})}{N}=\frac{H(M_{1}|Z_{1},Z_{2}^{N})}{N}\overset{\left(f\right)}{=}\frac{H(M_{1}|Z_{2}^{N})}{N}\\ & & =\frac{1}{N}\left(h\left(M_{1},Z_{2}^{N}\right)-h\left(Z_{2}^{N}\right)\right)\\ & & =\frac{1}{N}\left(h\left(M_{1},Z_{2}^{N},A_{2}^{*N}\right)-h\left(A_{2}^{*N}|M_{1},Z_{2}^{N}\right)-h\left(Z_{2}^{N}\right)\right)\\ & & =\frac{1}{N}\left(h\left(M_{1},A_{2}^{*N}\right)+h\left(Z_{2}^{N}|M_{1},A_{2}^{*N}\right)-h\left(A_{2}^{*N}|M_{1},Z_{2}^{N}\right)-h\left(Z_{2}^{N}\right)\right)\\ & & =\frac{1}{N}\left(h\left(M_{1},A_{2}^{*N}\right)-I\left(Z_{2}^{N};M_{1},A_{2}^{*N}\right)-h\left(A_{2}^{*N}|M_{1},Z_{2}^{N}\right)\right)\\ & & =\frac{1}{N}\left(h\left(M_{1},A_{2}^{*N}|Z_{2}^{N}\right)-h\left(A_{2}^{*N}|M_{1},Z_{2}^{N}\right)\right)\\ & & =\frac{1}{N}\left(h\left(A_{2}^{*N}|Z_{2}^{N}\right)+H\left(M_{1}|A_{2}^{*N},Z_{2}^{N}\right)-h\left(A_{2}^{*N}|M_{1},Z_{2}^{N}\right)\right)\\ & & =\frac{1}{N}\left(h\left(A_{2}^{*N}|Z_{2}^{N}\right)-h\left(A_{2}^{*N}|M_{1},Z_{2}^{N}\right)+h\left(A_{2}^{*N}\right)-h\left(A_{2}^{*N}\right)\right)\\ & & =\frac{1}{N}\left(h\left(A_{2}^{*N}\right)-I\left(Z_{2}^{N};A_{2}^{*N}\right)-h\left(A_{2}^{*N}|M_{1},Z_{2}^{N}\right)\right)\\ & & \overset{\left(g\right)}{=}\frac{1}{N}\left(H\left(M_{1},M_{1}^{*}\right)-I\left(Z_{2}^{N};A_{2}^{*N}\right)-h\left(A_{2}^{*N}|M_{1},Z_{2}^{N}\right)\right)\\ & & =\frac{1}{N}\left(\left(N-1\right)\left(R_{1}+R^{*}\right)-I\left(Z_{2}^{N};A_{2}^{*N}\right)-h\left(A_{2}^{*N}|M_{1},Z_{2}^{N}\right)\right)\\ & & \overset{\left(h\right)}{\geq }\frac{N-1}{N}\left(R_{1}+R^{*}\right)-\frac{I(Z_{2}^{N};A_{2}^{*N})}{N}-\frac{\delta (\varepsilon )}{N}\\ & & =\frac{N-1}{N}\left(R_{1}+R^{*}\right)-\frac{I(Z_{2}^{N};A_{2}^{*N})}{N-1}\frac{N-1}{N}-\frac{\delta (\varepsilon )}{N}, \end{array}$$

where (f) follows from the Markov chain $M_{1}\to Z_{2}^{N}\to Z_{1}$ and $Z_{2}^{N}=\left(Z_{2},Z_{3},\ldots ,Z_{N}\right)$, (g) is due to the fact that $A_{2}^{*N}$ is a deterministic function of $M_{1},M_{1}^{*}$, and (h) follows from Fano’s inequality when the codeword $A_{2}^{*N}$ is generated by Feinstein’s greedy construction [19], i.e., if

(46) $$\begin{array}{c} R^{*}\leq \frac{I(Z_{2}^{N};A_{2}^{*N})}{N-1}, \end{array}$$

given $M_{1}$, the eavesdropper’s decoding error probability $P_{ew}$ of $M_{1}^{*}$ is arbitrarily small ($P_{ew}\leq \epsilon$) as *N* tends to infinity, then using Fano’s inequality, we have

(47) $$\begin{array}{c} h\left(A_{2}^{*N}|M_{1},Z_{2}^{N}\right)\leq \delta \left(P_{ew}\right)\leq \delta \left(\varepsilon \right). \end{array}$$

Formula (45) indicates that *N* is sufficiently large, if

(48) $$\begin{array}{c} R^{*}\geq \frac{I(Z_{2}^{N};A_{2}^{*N})}{N-1}, \end{array}$$

we have

(49) $$\begin{array}{c} \frac{H(M_{1}|Z^{N})}{N}\geq R_{1}-\epsilon^{{}^{\prime }}, \end{array}$$

where $\epsilon^{{}^{\prime }}\to 0$ as N→∞.

From (46) and (48), we conclude that

(50) $$\begin{array}{c} R^{*}=\frac{I(Z_{2}^{N};A_{2}^{*N})}{N-1}. \end{array}$$

Substituting (50) into (38) and noting that the maximum of $R_{1}$ is achieved when N→∞, then we have

(51) $$\begin{array}{c} R_{1}\leq \lim_{N\to \infty }\left\{\frac{I\left(A_{2}^{*N};Y_{2}^{N}\right)-I\left(A_{2}^{*N};Z_{2}^{N}\right)}{N-1}\right\} \end{array}$$

**Lemma** **2.** *The fundamental limit of $R_{1}$ in (51) is upper bounded by*

(52) $$\begin{array}{ccc} & & R_{1}\leq \lim_{N\to \infty }\left\{\frac{I\left(A_{2}^{*N};Y_{2}^{N}\right)-I\left(A_{2}^{*N};Z_{2}^{N}\right)}{N-1}\right\}\\ & & \leq \frac{1}{2}\log\left(1+\frac{{\left(\sqrt{P_{A}}+\rho_{2}\sqrt{P}\right)}^{2}}{P\left(1-\rho_{1}^{2}-\rho_{2}^{2}\right)+{\left(\sigma_{w}+\rho_{1}\sqrt{P}\right)}^{2}+\sigma_{1}^{2}}\right)\\ & & -\frac{1}{2}\log\left(1+\frac{{\left(\sqrt{P_{A}}+\rho_{2}\sqrt{P}\right)}^{2}}{P\left(1-\rho_{1}^{2}-\rho_{2}^{2}\right)+{\left(\sigma_{w}+\rho_{1}\sqrt{P}\right)}^{2}+\sigma_{1}^{2}+\sigma_{2}^{2}}\right) \end{array}$$

**Proof.**  The *Proof of Lemma 2* is in Appendix B. □

The second part $\frac{H(M_{2}|Z^{N},M_{1})}{N}$ of (44) is bounded by

(53) $$\begin{array}{ccc} & & \frac{H(M_{2}|Z^{N},M_{1})}{N}\overset{\left(a\right)}{=}\frac{H(\theta |Z^{N},M_{1})}{N}\geq \frac{1}{N}H\left(\theta |Z^{N},\eta_{1,1},\ldots ,\eta_{1,N},\eta_{2,2},\ldots ,\eta_{2,N},W_{1}^{{}^{\prime }},\ldots ,W_{N}^{{}^{\prime }},M_{1},M_{1}^{*}\right)\\ & & \overset{\left(b\right)}{=}\frac{1}{N}H(\theta |\sqrt{12P^{{}^{\prime }}}\left(\theta -\frac{W_{1}^{{}^{\prime }}}{\sqrt{12P^{{}^{\prime }}}}+B\right)+W_{1}^{{}^{\prime }}+\eta_{1,1}+\eta_{2,1},\\ & & A_{2}^{*}+\sqrt{\frac{P^{{}^{\prime }}}{\alpha_{1}}}\varepsilon_{1}+W_{2}^{{}^{\prime }}+\eta_{1,2}+\eta_{2,2},A_{3}^{*}+f_{2,3}\left(\eta_{1,1},\eta_{1,2}\right)+W_{3}^{{}^{\prime }}+\eta_{1,3}+\eta_{2,3},\ldots ,\\ & & A_{N}^{*}+f_{2,N}\left(\eta_{1,1},\ldots ,\eta_{1,N-1}\right)+W_{N}^{{}^{\prime }}+\eta_{1,N}+\eta_{2,N},\eta_{1,1},\ldots ,\eta_{1,N},\eta_{2,2},\ldots ,\eta_{2,N},W_{1}^{{}^{\prime }},\ldots ,W_{N}^{{}^{\prime }},M_{1},M_{1}^{*})\\ & & =\frac{1}{N}H\left(\theta |\sqrt{12P^{{}^{\prime }}}\theta +\eta_{2,1},\eta_{1,1},\ldots ,\eta_{1,N},\eta_{2,2},\ldots ,\eta_{2,N},W_{1}^{{}^{\prime }},\ldots ,W_{N}^{{}^{\prime }},M_{1},M_{1}^{*}\right)\\ & & \overset{\left(c\right)}{=}\frac{1}{N}H\left(\theta |\sqrt{12P^{{}^{\prime }}}\theta +\eta_{2,1}\right)\\ & & \overset{\left(d\right)}{=}\frac{1}{N}\left\{H\left(\theta \right)-h\left(\sqrt{12P^{{}^{\prime }}}\theta +\eta_{2,1}\right)+h\left(\eta_{2,1}\right)\right\}\\ & & \overset{\left(e\right)}{\geq }R_{2}-\frac{1}{2N}\log\left(1+\frac{P^{{}^{\prime }}}{\sigma_{2}^{2}}\right) \end{array}$$

where (*a*) follows from the fact that there is a one-to-one mapping between $M_{2}$ and θ, (*b*) follows from (28), (30), (33) and (A5), (*c*) follows from the fact that θ and $\eta_{2,1}$ are independent of $\eta_{1,1},\ldots ,\eta_{1,N},\eta_{2,2},\ldots ,\eta_{2,N},W_{1}^{{}^{\prime }},\ldots ,W_{N}^{{}^{\prime }},M_{1},M_{1}^{*}$, (*d*) follows from the fact that θ and $\eta_{2,1}$ are independent of each other, and (*e*) follows from $H\left(\theta \right)=NR_{2}$, and the variance of θ equals $\frac{1}{12}$ as *N* tends to infinity.

Substituting (49) and (53) into (44), we have

(54) $$\begin{array}{ccc} & & \frac{H(M|Z^{N})}{N}=\frac{H\left(M_{1}|Z^{N}\right)+H\left(M_{2}|Z^{N},M_{1}\right)}{N}\\ & & \geq R_{1}-\epsilon^{{}^{\prime }}+R_{2}-\frac{1}{2N}\log\left(1+\frac{P^{{}^{\prime }}}{\sigma_{2}^{2}}\right)\\ & & =R-(\epsilon^{{}^{\prime }}+\frac{1}{2N}\log\left(1+\frac{P^{{}^{\prime }}}{\sigma_{2}^{2}}\right)) \end{array}$$

where $R=R_{1}+R_{2}$. Choosing sufficiently large *N*, $\frac{H(M|Z^{N})}{N}\geq R-\epsilon$ is satisfied. Finally, combining (43) and (52), the lower bound in *Theorem 1* is derived, and the proof is completed.

## 5. Conclusions and Future Work

This paper shows a new lower bound on the secrecy capacity of the AD-DP-WTC with noiseless feedback by proposing a novel two-step hybrid feedback scheme. The numerical result shows that when the noise variance $\sigma_{w}^{2}$ is large enough, our new feedback scheme performs better than the existing one in the literature. Moreover, when the eavesdropper’s channel noise variance is fairly large, our new feedback scheme is almost optimal (the new lower bound almost equals the upper bound). Possible future work could be to extend the proposed feedback scheme to the multiple-access situation.

## Data Availability

Not applicable.

## Appendix A. Proof of Lemma 1

From (32), we know that $\varepsilon_{1}=\frac{\eta_{1,1}}{\sqrt{12P^{\prime }}}$ and $\alpha_{1}=Var\left(\varepsilon_{1}\right)$, and hence $\alpha_{1}=E{\left(\frac{\eta_{1,1}}{\sqrt{12P^{\prime }}}\right)}^{2}=\frac{\sigma_{1}^{2}}{12P^{\prime }}$.

In the same way, since $\varepsilon_{2}=\varepsilon_{1}-\frac{E(Y_{2}^{\prime \prime }\varepsilon_{1})}{E{\left(Y_{2}^{\prime \prime }\right)}^{2}}Y_{2}^{\prime \prime }$ and $\alpha_{2}=Var\left(\varepsilon_{2}\right)$, we have

(A1) $$\begin{array}{ccc} & & \alpha_{2}=Var\left(\varepsilon_{2}\right)=E{\left(\varepsilon_{1}-\frac{E(Y_{2}^{\prime \prime }\varepsilon_{1})}{E{\left(Y_{2}^{\prime \prime }\right)}^{2}}Y_{2}^{\prime \prime }\right)}^{2}=E{\left(\varepsilon_{1}\right)}^{2}-\frac{E{\left(Y_{2}^{\prime \prime }\varepsilon_{1}\right)}^{2}}{E{\left(Y_{2}^{\prime \prime }\right)}^{2}}\\ & & =\alpha_{1}-\frac{\alpha_{1}P^{\prime }}{P^{\prime }+\sigma_{1}^{2}}=\alpha_{1}\frac{\sigma_{1}^{2}}{P^{\prime }+\sigma_{1}^{2}}, \end{array}$$

where $Y_{2}^{\prime \prime }=V_{2}+\eta_{1,2}=\sqrt{\frac{P^{\prime }}{\alpha_{1}}}\varepsilon_{1}+\eta_{1,2}$.

For 3≤k≤N,

(A2) $$\begin{array}{ccc} & & \alpha_{k-1}=Var\left(\varepsilon_{k-1}\right)\overset{\left(a\right)}{=}E{\left(\varepsilon_{k-2}-\frac{E(Y_{k-1}^{\prime \prime }\varepsilon_{k-2})}{E{\left(Y_{k-1}^{\prime \prime }\right)}^{2}}Y_{k-1}^{\prime \prime }\right)}^{2}=E{\left(\varepsilon_{k-2}\right)}^{2}-\frac{E{\left(Y_{k-1}^{\prime \prime }\varepsilon_{k-2}\right)}^{2}}{E{\left(Y_{k-1}^{\prime \prime }\right)}^{2}}\\ & & =\alpha_{k-2}-\frac{\alpha_{k-2}P^{\prime }}{P^{\prime }+\sigma_{1}^{2}}=\alpha_{k-2}\frac{\sigma_{1}^{2}}{P^{\prime }+\sigma_{1}^{2}}=\alpha_{1}{\left(\frac{\sigma_{1}^{2}}{P^{\prime }+\sigma_{1}^{2}}\right)}^{k-2}, \end{array}$$

where (a) follows from (34), and $Y_{k-1}^{\prime \prime }=V_{k-1}+\eta_{1,k-1}=\sqrt{\frac{P^{\prime }}{\alpha_{k-2}}}\varepsilon_{k-2}+\eta_{1,k-1}$.

Now, it remains to further compute the second part of *Lemma 1*. According to (35), we have $V_{k}=\sqrt{\frac{P^{\prime }}{\alpha_{k-1}}}\varepsilon_{k-1}$, and it can be further expressed as

(A3) $$\begin{array}{ccc} & & V_{k}=\sqrt{\frac{P^{\prime }}{\alpha_{k-1}}}\varepsilon_{k-1}\overset{\left(b\right)}{=}\sqrt{\frac{P^{\prime }}{\alpha_{k-1}}}\left\{\varepsilon_{k-2}-\frac{E\left(V_{k-1}+\eta_{1,k-1}\right)\varepsilon_{k-2}}{E{\left(V_{k-1}+\eta_{1,k-1}\right)}^{2}}\left(V_{k-1}+\eta_{1,k-1}\right)\right\}\\ & & =\sqrt{\frac{P^{\prime }}{\alpha_{k-2}}}\sqrt{\frac{\alpha_{k-2}}{\alpha_{k-1}}}\left\{\varepsilon_{k-2}-\frac{E\left(V_{k-1}+\eta_{1,k-1}\right)\varepsilon_{k-2}}{E{\left(V_{k-1}+\eta_{1,k-1}\right)}^{2}}\left(V_{k-1}+\eta_{1,k-1}\right)\right\}\\ & & =\sqrt{\frac{\alpha_{k-2}}{\alpha_{k-1}}}V_{k-1}-\sqrt{\frac{P^{\prime }}{\alpha_{k-1}}}\frac{\sqrt{\alpha_{k-2}P^{\prime }}}{P^{\prime }+\sigma_{1}^{2}}\left(V_{k-1}+\eta_{1,k-1}\right)\\ & & =\sqrt{\frac{\alpha_{k-2}}{\alpha_{k-1}}}\frac{\sigma_{1}^{2}}{P^{\prime }+\sigma_{1}^{2}}V_{k-1}-\sqrt{\frac{\alpha_{k-2}}{\alpha_{k-1}}}\frac{P^{\prime }}{P^{\prime }+\sigma_{1}^{2}}\eta_{1,k-1}, \end{array}$$

where (b) follows from (34), and $Y_{k-1}^{\prime \prime }=V_{k-1}+\eta_{1,k-1}=\sqrt{\frac{P^{\prime }}{\alpha_{k-2}}}\varepsilon_{k-2}+\eta_{1,k-1}$.

Therefore, we conclude that

(A4) $$\begin{array}{ccc} & & V_{3}=\sqrt{\frac{\alpha_{1}}{\alpha_{2}}}\frac{\sigma_{1}^{2}}{P^{\prime }+\sigma_{1}^{2}}V_{2}-\sqrt{\frac{\alpha_{1}}{\alpha_{2}}}\frac{P^{\prime }}{P^{\prime }+\sigma_{1}^{2}}\eta_{1,2},\\ & & V_{4}=\sqrt{\frac{\alpha_{2}}{\alpha_{3}}}\frac{\sigma_{1}^{2}}{P^{\prime }+\sigma_{1}^{2}}V_{3}-\sqrt{\frac{\alpha_{2}}{\alpha_{3}}}\frac{P^{\prime }}{P^{\prime }+\sigma_{1}^{2}}\eta_{1,3}\\ & & =\sqrt{\frac{\alpha_{1}}{\alpha_{3}}}{\left(\frac{\sigma_{1}^{2}}{P^{\prime }+\sigma_{1}^{2}}\right)}^{2}V_{2}-\sum_{i=1}^{2}\sqrt{\frac{\alpha_{i}}{\alpha_{3}}}\frac{P^{\prime }{\left(\sigma_{1}^{2}\right)}^{2-i}}{{\left(P^{\prime }+\sigma_{1}^{2}\right)}^{4-(i+1)}}\eta_{1,i+1}\\ & & \;\;\;\vdots \\ & & V_{k}=\sqrt{\frac{\alpha_{1}}{\alpha_{k-1}}}{\left(\frac{\sigma_{1}^{2}}{P^{\prime }+\sigma_{1}^{2}}\right)}^{k-2}V_{2}-\sum_{i=1}^{k-2}\sqrt{\frac{\alpha_{i}}{\alpha_{k-1}}}\frac{P^{\prime }{\left(\sigma_{1}^{2}\right)}^{k-2-i}}{{\left(P^{\prime }+\sigma_{1}^{2}\right)}^{k-(i+1)}}\eta_{1,i+1}\\ & & \overset{\left(c\right)}{=}\frac{1}{\sqrt{12\alpha_{k-1}}}{\left(\frac{\sigma_{1}^{2}}{P^{\prime }+\sigma_{1}^{2}}\right)}^{k-2}\eta_{1,1}-\sum_{i=1}^{k-2}\sqrt{\frac{\alpha_{i}}{\alpha_{k-1}}}\frac{P^{\prime }{\left(\sigma_{1}^{2}\right)}^{k-2-i}}{{\left(P^{\prime }+\sigma_{1}^{2}\right)}^{k-(i+1)}}\eta_{1,i+1} \end{array}$$

where (c) follows from $V_{2}=\sqrt{\frac{P^{\prime }}{\alpha_{1}}}\varepsilon_{1}=\sqrt{\frac{P^{\prime }}{\alpha_{1}}}\frac{\eta_{1,1}}{\sqrt{12P^{\prime }}}$. From (A4) and (33), we see that $V_{k}$ is a function of $\left(\eta_{1,1},\eta_{1,2},\ldots ,\eta_{1,k-1}\right)$, i.e.,

(A5) $$\begin{array}{c} V_{k}=f_{2,k}\left(\eta_{1,1},\eta_{1,2},\ldots ,\eta_{1,k-1}\right),\;\;2\leq k\leq N. \end{array}$$

Finally, according to (A2) and (A4), we complete the proof of *Lemma 1*.

## Appendix B. Proof of Lemma 2

Since

(A6) $$\begin{array}{ccc} & & \lim_{N\to \infty }\frac{I\left(A_{2}^{*N};Y_{2}^{N}\right)-I\left(A_{2}^{*N};Z_{2}^{N}\right)}{N-1}=\lim_{N\to \infty }\frac{h\left(Y_{2}^{N}\right)-h\left(Y_{2}^{N}|A_{2}^{*N}\right)-h\left(Z_{2}^{N}\right)+h\left(Z_{2}^{N}|A_{2}^{*N}\right)}{N-1}\\ & & \overset{\left(a\right)}{=}\lim_{N\to \infty }\left\{\frac{h(Y_{2}^{N})}{N-1}-\frac{h(Z_{2}^{N})}{N-1}-\frac{h(\eta_{1,2}^{{}^{\prime }N})}{N-1}+\frac{h(\eta_{1,2}^{{}^{\prime }N}+\eta_{2,2}^{N})}{N-1}\right\}, \end{array}$$

where (a) is due to the fact that $Y_{2}^{N}=A_{2}^{*N}+\eta_{1,2}^{{}^{\prime }N},\;\;Z_{2}^{N}=Y_{2}^{N}+\eta_{2,2}^{N}$, $\eta_{1,2}^{{}^{\prime }N}=V_{2}^{N}+W_{2}^{{}^{\prime }N}+\eta_{1,2}^{N}$ and $A_{2}^{*N}$ is independent of $\eta_{1,2}^{{}^{\prime }N},\eta_{2,2}^{N}$, and $\eta_{1,2}^{{}^{\prime }N}=\left(\eta_{1,2}^{\prime },\eta_{1,3}^{\prime },\ldots ,\eta_{1,N}^{\prime }\right),\;\;\eta_{2,2}^{N}=\left(\eta_{2,2},\eta_{2,3},\ldots ,\eta_{2,N}\right)$.

For the part $\frac{h(Y_{2}^{N})}{N-1}-\frac{h(Z_{2}^{N})}{N-1}$ of (A6), we have

(A7) $$\begin{array}{ccc} & & \lim_{N\to \infty }\left\{\frac{h(Y_{2}^{N})}{N-1}-\frac{h(Z_{2}^{N})}{N-1}\right\}=\lim_{N\to \infty }\left\{\frac{h(Y_{2}^{N})}{N-1}-\frac{h(Y_{2}^{N}+\eta_{2,2}^{N})}{N-1}\right\}\\ & & \overset{\left(b\right)}{\leq }\lim_{N\to \infty }\left\{\frac{h(Y_{2}^{N})}{N-1}-\frac{1}{2}\log\left(2^{\frac{2h(Y_{2}^{N})}{N-1}}+2\pi e\sigma_{2}^{2}\right)\right\}\\ & & \overset{\left(c\right)}{\leq }\frac{1}{2}\log2\pi e\left(P^{*}+P^{\prime }+\sigma_{w}^{{}^{\prime }2}+\sigma_{1}^{2}\right)-\frac{1}{2}\log2\pi e\left(P^{*}+P^{\prime }+\sigma_{w}^{{}^{\prime }2}+\sigma_{1}^{2}+\sigma_{2}^{2}\right), \end{array}$$

where (b) follows from the entropy power inequality, (c) follows from $\frac{h(Y_{2}^{N})}{N-1}-\frac{1}{2}\log\left(2^{\frac{2h(Y_{2}^{N})}{N-1}}+2\pi e\sigma_{2}^{2}\right)$ increasing while $\frac{h(Y_{2}^{N})}{N-1}$ is increasing and

(A8) $$\begin{array}{ccc} & & \frac{h(Y_{2}^{N})}{N-1}\leq \frac{1}{N-1}\sum_{i=2}^{N}h\left(Y_{i}\right)\leq \frac{1}{N-1}\sum_{i=2}^{N}\frac{1}{2}\log2\pi eE\left(Y_{i}^{2}\right)\\ & & =\frac{N}{N-1}\frac{1}{N}\sum_{i=2}^{N}\frac{1}{2}\log2\pi eE\left(Y_{i}^{2}\right)\\ & & \leq \frac{1}{2}\frac{N}{N-1}\log2\pi e\left(P^{*}+P^{\prime }+\sigma_{w}^{{}^{\prime }2}+\sigma_{1}^{2}\right), \end{array}$$

where $P^{*}=P_{A}+\rho_{2}^{2}P+2\rho_{2}\sqrt{PP_{A}},\;\;\;P^{\prime }=P\left(1-\rho_{1}^{2}-\rho_{2}^{2}\right),\;\;\;\sigma_{w}^{{}^{\prime }2}={\left(\sigma_{w}+\rho_{1}\sqrt{P}\right)}^{2}$, and the last inequality of (A8) is from Jensen’s inequality.

Based on the joint differential entropy, for the part $\frac{h(\eta_{1,2}^{{}^{\prime }N})}{N-1}$ of (A6), we have

(A9) $$\begin{array}{c} \lim_{N\to \infty }h\left(\eta_{1,2}^{{}^{\prime }N}\right)=\lim_{N\to \infty }\frac{1}{2}\log{\left(2\pi e\right)}^{N-1}\left|K\right|, \end{array}$$

where *K* is a covariance matrix, and

(A10) $$\begin{array}{c} \left|K\right|={\left|\begin{array}{cccc} E(\eta_{1,2}^{\prime }\eta_{1,2}^{\prime }) & E(\eta_{1,2}^{\prime }\eta_{1,3}^{\prime }) & \cdots & E(\eta_{1,2}^{\prime }\eta_{1,N}^{\prime })\\ E(\eta_{1,3}^{\prime }\eta_{1,2}^{\prime }) & E(\eta_{1,3}^{\prime }\eta_{1,3}^{\prime }) & \cdots & E(\eta_{1,3}^{\prime }\eta_{1,N}^{\prime })\\ \vdots & \vdots & \cdots & \vdots \\ E(\eta_{1,N}^{\prime }\eta_{1,2}^{\prime }) & E(\eta_{1,N}^{\prime }\eta_{1,3}^{\prime }) & \cdots & E(\eta_{1,N}^{\prime }\eta_{1,N}^{\prime }) \end{array}\right|}_{\left(N-1)\times (N-1\right)} \end{array}$$

with

(A11) $$\begin{array}{c} E\left(\eta_{1,k}^{\prime }\eta_{1,k}^{\prime }\right)=P^{\prime }+\sigma_{w}^{{}^{\prime }2}+\sigma_{1}^{2},\;\;\;2\leq k\leq N \end{array}$$

(A12) $$\begin{array}{ccc} & & E\left(\eta_{1,k}^{\prime }\eta_{1,j}^{\prime }\right)=E\left(V_{k}+W_{k}^{\prime }+\eta_{1,k}\right)\left(V_{j}+W_{j}^{\prime }+\eta_{1,j}\right)\\ & & =E\left(V_{k}V_{j}\right)+E\left(\eta_{1,k}V_{j}\right),\;\;\;2\leq k\leq N,\;\;\;k<j\leq N. \end{array}$$

According to (A4), we have

(A13) $$\begin{array}{ccc} & & V_{k}=\sqrt{\frac{\alpha_{1}}{\alpha_{k-1}}}{\left(\frac{\sigma_{1}^{2}}{P^{\prime }+\sigma_{1}^{2}}\right)}^{k-2}V_{2}-\sum_{i=1}^{k-2}\sqrt{\frac{\alpha_{i}}{\alpha_{k-1}}}\frac{P^{\prime }{\left(\sigma_{1}^{2}\right)}^{k-2-i}}{{\left(P^{\prime }+\sigma_{1}^{2}\right)}^{k-(i+1)}}\eta_{1,i+1},\\ & & V_{j}=\sqrt{\frac{\alpha_{1}}{\alpha_{j-1}}}{\left(\frac{\sigma_{1}^{2}}{P^{\prime }+\sigma_{1}^{2}}\right)}^{j-2}V_{2}-\sum_{i=1}^{j-2}\sqrt{\frac{\alpha_{i}}{\alpha_{j-1}}}\frac{P^{\prime }{\left(\sigma_{1}^{2}\right)}^{j-2-i}}{{\left(P^{\prime }+\sigma_{1}^{2}\right)}^{j-(i+1)}}\eta_{1,i+1}\\ & & =\sqrt{\frac{\alpha_{1}}{\alpha_{j-1}}}{\left(\frac{\sigma_{1}^{2}}{P^{\prime }+\sigma_{1}^{2}}\right)}^{j-2}V_{2}-\sum_{i=1}^{k-2}\sqrt{\frac{\alpha_{i}}{\alpha_{j-1}}}\frac{P^{\prime }{\left(\sigma_{1}^{2}\right)}^{j-2-i}}{{\left(P^{\prime }+\sigma_{1}^{2}\right)}^{j-(i+1)}}\eta_{1,i+1}\\ & & -\sqrt{\frac{\alpha_{k-1}}{\alpha_{j-1}}}\frac{P^{\prime }{\left(\sigma_{1}^{2}\right)}^{j-k-1}}{{\left(P^{\prime }+\sigma_{1}^{2}\right)}^{j-k}}\eta_{1,k}-\sum_{i=k}^{j-2}\sqrt{\frac{\alpha_{i}}{\alpha_{j-1}}}\frac{P^{\prime }{\left(\sigma_{1}^{2}\right)}^{j-2-i}}{{\left(P^{\prime }+\sigma_{1}^{2}\right)}^{j-(i+1)}}\eta_{1,i+1}, \end{array}$$

Based on (A13), we can conclude that

(A14) $$\begin{array}{ccc} & & E\left(\eta_{1,k}V_{j}\right)=-E\left(\eta_{1,k}\sqrt{\frac{\alpha_{k-1}}{\alpha_{j-1}}}\frac{P^{\prime }{\left(\sigma_{1}^{2}\right)}^{j-k-1}}{{\left(P^{\prime }+\sigma_{1}^{2}\right)}^{j-k}}\eta_{1,k}\right)\\ & & =-\sqrt{\frac{\alpha_{k-1}}{\alpha_{j-1}}}\frac{P^{\prime }{\left(\sigma_{1}^{2}\right)}^{j-k}}{{\left(P^{\prime }+\sigma_{1}^{2}\right)}^{j-k}}\overset{\left(d\right)}{=}-P^{\prime }{\left(\frac{\sigma_{1}^{2}}{P^{\prime }+\sigma_{1}^{2}}\right)}^{\frac{j-k}{2}}, \end{array}$$

where (d) follows from (A2). For $E(V_{k}V_{j})$, we have

(A15) $$\begin{array}{ccc} & & E\left(V_{k}V_{j}\right)\overset{\left(e\right)}{=}E\{\left[\sqrt{\frac{\alpha_{1}}{\alpha_{k-1}}}{\left(\frac{\sigma_{1}^{2}}{P^{\prime }+\sigma_{1}^{2}}\right)}^{k-2}V_{2}-\sum_{i=1}^{k-2}\sqrt{\frac{\alpha_{i}}{\alpha_{k-1}}}\frac{P^{\prime }{\left(\sigma_{1}^{2}\right)}^{k-2-i}}{{\left(P^{\prime }+\sigma_{1}^{2}\right)}^{k-(i+1)}}\eta_{1,i+1}\right]\\ & & \times [\sqrt{\frac{\alpha_{1}}{\alpha_{j-1}}}{\left(\frac{\sigma_{1}^{2}}{P^{\prime }+\sigma_{1}^{2}}\right)}^{j-2}V_{2}-\sum_{i=1}^{k-2}\sqrt{\frac{\alpha_{i}}{\alpha_{j-1}}}\frac{P^{\prime }{\left(\sigma_{1}^{2}\right)}^{j-2-i}}{{\left(P^{\prime }+\sigma_{1}^{2}\right)}^{j-(i+1)}}\eta_{1,i+1}]\}\\ & & =P^{\prime }\sqrt{\frac{\alpha_{1}}{\alpha_{k-1}}}\sqrt{\frac{\alpha_{1}}{\alpha_{j-1}}}{\left(\frac{\sigma_{1}^{2}}{P^{\prime }+\sigma_{1}^{2}}\right)}^{k+j-4}+\sum_{i=1}^{k-2}\sqrt{\frac{\alpha_{i}}{\alpha_{k-1}}}\sqrt{\frac{\alpha_{i}}{\alpha_{j-1}}}\frac{{\left(P^{\prime }\right)}^{2}{\left(\sigma_{1}^{2}\right)}^{k+j-3-2i}}{{\left(P^{\prime }+\sigma_{1}^{2}\right)}^{k+j-2(i+1)}}\\ & & \overset{\left(f\right)}{=}P^{\prime }{\left(\frac{\sigma_{1}^{2}}{P^{\prime }+\sigma_{1}^{2}}\right)}^{\frac{k+j-4}{2}}+\sum_{i=1}^{k-2}\frac{{\left(P^{\prime }\right)}^{2}}{\sigma_{1}^{2}}{\left(\frac{\sigma_{1}^{2}}{P^{\prime }+\sigma_{1}^{2}}\right)}^{\frac{k+j-2(i+1)}{2}}\\ & & =P^{\prime }{\left(\frac{\sigma_{1}^{2}}{P^{\prime }+\sigma_{1}^{2}}\right)}^{\frac{j-k}{2}}, \end{array}$$

where (e) follows from (A13), and (f) follows from (A2).

Hence, (A12) can be further calculated by

(A16) $$\begin{array}{c} E\left(\eta_{1,k}^{\prime }\eta_{1,j}^{\prime }\right)=E\left(V_{k}V_{j}\right)+E\left(\eta_{1,k}V_{j}\right)\overset{\left(g\right)}{=}0,\;\;\;2\leq k\leq N,\;\;\;k<j\leq N, \end{array}$$

where (g) follows from (A15) and (A14).

According to (A16) and (A11),

(A17) $$\begin{array}{c} \left|K\right|={\left|\begin{array}{cccc} P^{\prime }+\sigma_{w}^{{}^{\prime }2}+\sigma_{1}^{2} & 0 & \cdots & 0\\ 0 & P^{\prime }+\sigma_{w}^{{}^{\prime }2}+\sigma_{1}^{2} & \cdots & 0\\ \vdots & \vdots & \cdots & \vdots \\ 0 & 0 & \cdots & P^{\prime }+\sigma_{w}^{{}^{\prime }2}+\sigma_{1}^{2} \end{array}\right|}_{\left(N-1)\times (N-1\right)} \end{array}$$

Therefore, we can obtain that

(A18) $$\begin{array}{c} \lim_{N\to \infty }\frac{1}{N-1}h\left(\eta_{1,2}^{{}^{\prime }N}\right)=\lim_{N\to \infty }\frac{1}{2(N-1)}\log{\left(2\pi e\right)}^{N-1}\left|K\right|=\frac{1}{2}\log2\pi e\left(P^{\prime }+\sigma_{w}^{{}^{\prime }2}+\sigma_{1}^{2}\right). \end{array}$$

Analogously, we have

(A19) $$\begin{array}{c} \lim_{N\to \infty }\frac{1}{N-1}h\left(\eta_{1,2}^{{}^{\prime }N}+\eta_{2,2}^{N}\right)=\frac{1}{2}\log2\pi e\left(P^{\prime }+\sigma_{w}^{{}^{\prime }2}+\sigma_{1}^{2}+\sigma_{2}^{2}\right). \end{array}$$

Substituting (A7), (A18) and (A19) into (A6), we prove that

(A20) $$\begin{array}{ccc} & & \lim_{N\to \infty }\frac{I\left(A_{2}^{*N};Y_{2}^{N}\right)-I\left(A_{2}^{*N};Z_{2}^{N}\right)}{N-1}\\ & & \leq \frac{1}{2}\log\left(1+\frac{{\left(\sqrt{P_{A}}+\rho_{2}\sqrt{P}\right)}^{2}}{P\left(1-\rho_{1}^{2}-\rho_{2}^{2}\right)+{\left(\sigma_{w}+\rho_{1}\sqrt{P}\right)}^{2}+\sigma_{1}^{2}}\right)\\ & & -\frac{1}{2}\log\left(1+\frac{{\left(\sqrt{P_{A}}+\rho_{2}\sqrt{P}\right)}^{2}}{P\left(1-\rho_{1}^{2}-\rho_{2}^{2}\right)+{\left(\sigma_{w}+\rho_{1}\sqrt{P}\right)}^{2}+\sigma_{1}^{2}+\sigma_{2}^{2}}\right), \end{array}$$

which completes the proof.
